# The effects of crystalloid versus synthetic colloid *in vitro* on immune cells, co-cultured with mouse splenocytes

**DOI:** 10.1038/s41598-018-22981-8

**Published:** 2018-03-19

**Authors:** Seung Hyun Lee, Eun-Hye Seo, Hyun Jun Park, Chung-Sik Oh, Cho Long Kim, Sewon Park, Seong-Hyop Kim

**Affiliations:** 1 Department of Microbiology, Konkuk University School of Medicine, Seoul, Korea; 2 Research Institute of Medical Science, Konkuk University School of Medicine, Seoul, Korea; 3 BK21 Plus, Department of Cellular and Molecular Medicine, Konkuk University School of Medicine, Seoul, Korea; 4 Department of Anesthesiology and Pain medicine, Konkuk University Medical Centre, Konkuk University School of Medicine, Seoul, Korea; 5 Department of Infection and Immunology, Konkuk University School of Medicine, Seoul, Korea

## Abstract

This study assessed the effects of crystalloid versus synthetic colloid *in vitro* on immune cells co-cultured with mouse splenocytes. Mouse splenocytes were co-cultured with three different types of fluid: Plasma solution-A^®^ (CJ HealthCare, Seoul, Korea; the crystalloid group); Tetraspan 6%^®^ (B. Braun Medical, Melsungen, Germany; the Colloid-T group); and Volulyte 6%^®^ (Fresenius Kabi, Bad Homburg vor dér-Höhe, Germany; Colloid-V group). To evaluate the acquired immune response, cluster of differentiation (CD) 4^+^ T cells and CD8^+^ T cells were measured. To evaluate the innate immune response, neutrophils were measured. The frequencies of CD4^+^ and CD8^+^ T cells did not differ significantly among the three groups on day 1 or 3. However, the frequencies of CD4^+^ and CD8^+^ T cells in the two synthetic colloid groups were significantly higher than those in the crystalloid group on day 7. On day 1, the frequency of neutrophils was significantly lower in the two synthetic colloid groups, compared with the crystalloid group. However, the values on the other days were similar among all three groups. In conclusion, crystalloid had a limited effect on the immune response; on the other hand, synthetic colloid increased the acquired immune response, although it temporarily inhibited the innate immune response.

## Introduction

Perioperative fluid therapy is essential for maintaining tissue perfusion and oxygenation. Classically, synthetic colloid has been used to support the circulation and replace the blood loss, because it is theoretically superior to crystalloid in increasing the intravascular volume. However, recent meta-analyses have shown that there is no significant benefit to fluid therapy, using synthetic colloid^1–4^. Rather, the use of hydroxyethyl starch in septic patients has been associated with acute kidney injury with increased mortality^5^. Conversely, balanced synthetic colloid did not cause significant changes in renal function with blood loss in patients undergoing cardiac surgery^6^. Raiman *et al*. concluded that the data are insufficient to identify a difference in outcomes between crystalloid and synthetic colloid^7^. Therefore, we sought to investigate the effects of fluid therapy, using crystalloid versus synthetic colloid on immunity to verify the clinical outcomes.

We hypothesized that fluid therapy using crystalloid produced a lower immune response than that using synthetic colloid. Therefore, this study compared the effects of crystalloid versus synthetic colloid *in vitro* on immune cells co-cultured with mouse splenocytes.

## Materials and Methods

The experiments were approved by the Institutional Animal Care and Use Committee (IACUC) of Konkuk University (approval number: KU17043) on March 22 2017, and were conducted at the Konkuk University Laboratory Animal Research Center. The experiments were performed in accordance with relevant guidelines and regulations.

A 6-week-old male Balb/c mouse weighing 20 g was used in the experiment. The animal was quarantined for 2 weeks to confirm that it was pathogen-free before being sacrificed by cervical dislocation. To evaluate the acquired immune response, cluster of differentiation (CD) 4^+^ and CD8^+^ T cells in the spleen after cell culture with crystalloid and synthetic colloid were evaluated by flow cytometry. The chemokines produced by CD4^+^ and CD8^+^ T cells were measured. To evaluate the innate immune response, neutrophils producing reactive oxygen species (ROS) were measured.

### Splenocyte isolation

After euthanasia, the mouse abdomen was dissected, and the spleen was removed. The spleen was ground in 10 mL phosphate-buffered saline (PBS), using a cell strainer with 70-μm pores (SPL life Sciences, Pochoen, Korea). The filtered cells were suspended in PBS, and the splenocytes were separated from red blood cells and PBS using Ficoll density-gradient centrifugation at 2,400 rpm for 20 min at room temperature. After separation, the splenocytes were isolated using serum-separating tubes. The separated splenocytes were washed with 13 mL PBS and centrifuged at 1,500 rpm for 5 min. The cell pellet was resuspended in 2 mL Roswell Park Memorial Institute medium containing 10% fetal bovine serum (HyClone, Logan, UT, USA) and 1% penicillin/streptomycin (Life Technologies, Carlsbad, CA, USA), and the cells were counted.

### Cell culture

Three study groups were evaluated: the Crystalloid group and the two synthetic colloid groups (Colloid-T and Colloid-V groups). Plasma solution-A^®^ (CJ HealthCare, Seoul, Korea) was used for the Crystalloid group, Tetraspan 6%^®^ (B. Braun Medical, Melsungen, Germany) for the Colloid-T group, and Volulyte 6%^®^ (Fresenius Kabi, Bad Homburg vor dér-Höhe, Germany) for the Colloid-V group. Table 1 summarizes the compositions of the fluids. For each group, 5 × 10^5^ isolated splenocytes in 500 μL medium were added to the wells of culture plates. The culture plates were incubated at 37 °C for 1, 3, or 7 days.

**Table 1:** Composition of the different types of fluids in the study.

Parameter	Plasma solution-A^®^	Tetraspan 6%^®^	Volulyte 6%^®^
Hydroxyethyl starch (g/L)	—	60	60
pH	6.5–8.0	5.6–6.4	5.7–6.5
Osmolarity (mOsmol/L)	295	296	286.5
Na^+^ (mEq/L)	140	140	137
K^+^ (mEq/L)	5.0	4.0	4.0
Ca^2+^ (mEq/L)	—	2.5	—
Mg^2+^ (mEq/L)	3.0	1.0	1.5
Cl^−^ (mEq/L)	98	118	110
Acetate (mEq/L)	27	24	34
Gluconate (mEq/L)	23	—	—
Malate (mEq/L)	—	5	—

### Immunofluorescence staining of immune cells

After culturing, the supernatant was aliquoted into two 5 mL round-bottom tubes (Corning, Korea) and washed with fluorescence activated cell sorter (FACS) buffer (0.1% bovine serum albumin and 0.05% sodium azide in 1× PBS solution). After washing, the pellet in one round-bottom tube was stained with allophycocyanin (APC)-conjugated CD4 monoclonal antibody to evaluate CD4^+^ T cells. The pellet in the other round-bottom tube was stained with peridinin chlorophyll protein complex (PerCP) CD3 and APC-conjugated CD8 to monitor CD3^+^CD8^+^ T cells. The two tubes were incubated for 30 min at room temperature in the dark. Then, the immunostained cells in the tubes were washed with FACS buffer, resuspended, and analyzed using the FACS Accuri C6 (BD Biosciences, Seoul, Korea).

### Chemokines produced by immune cells

After culturing, the immune cells in the round-bottom tube were stained with monoclonal antibodies targeting phycoerythrin (PE) CC-type chemokine receptor 6 (CCR6) and fluorescein isothiocyanate (FITC) CXC chemokine receptor 3 (CXCR3) to evaluate CD4^+^ and CD8^+^ T cells, respectively. The stained cells were incubated for 30 min at room temperature in the dark and then washed with FACS buffer, resuspended, and analyzed using the FACS Accuri C6 (BD Biosciences).

### Cytokines produced by immune cells

After culturing, conditioned medium was collected to examine cytokines. The cytokine levels were determined using mouse enzyme-linked immunosorbent assay kits (R&D Systems, Minneapolis, MN, USA) for interferon (IFN)-γ, interleukin-2, and tumor necrosis factor (TNF)-α for CD4^+^ T cells, and IFN-γ for CD8^+^ T cells, respectively, using the Luminex 100 detection system (Luminex, Austin, TX, USA).

### ROS-producing neutrophils

After culturing, the cell pellet in the round-bottom tube was stained with FITC-anti mouse Ly-6G, 2′,7′-dichlorofluorescein diacetate (Life Technologies) to detect ROS in neutrophils. The staining was performed for 30 min in the dark at room temperature. The neutrophils were analyzed using FlowJo software (Tree Star, Ashland, OR, USA).

### Statistical analysis

The measured immune cells were compared by unpaired *t-*test, using GraphPad Prism 5 (GraphPad Software Inc., La Jolla, CA, USA). All data are expressed as means ± standard deviation. A *p*-value less than 0.05 was considered to indicate statistical significance.

## Results

**Figure 1:**
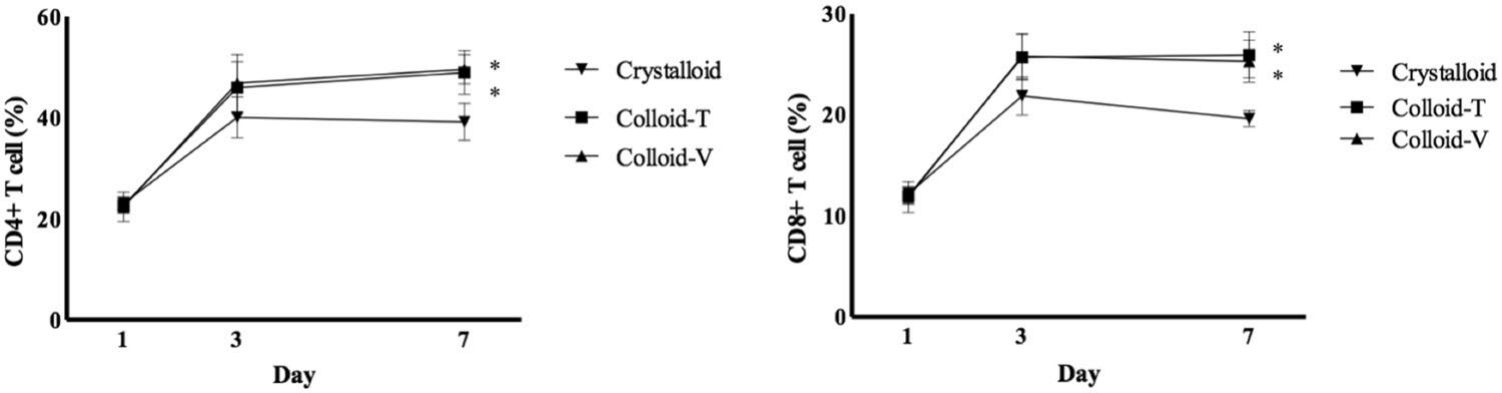
The expression of cluster of differentiation (CD) 4^+^ and CD8^+^ T cells. ^*^p < 0.05 compared with Crystalloid group.

The frequencies of CD4^+^ and CD8^+^ T cells did not differ significantly among the three groups on days 1 and 3. However, the numbers of CD4^+^ and CD8^+^ T cells on day 7 were significantly higher in the Colloid-T and Colloid-V groups than the Crystalloid group [CD4^+^ cells: 49.52 ± 9.17% in the Colloid T group (*p* < 0.05) and 49.20 ± 6.86% in the Colloid V group (*p* < 0.05) *vs*. 35.92 ± 4.56% in the Crystalloid group; CD8^+^ cells: 26.70 ± 4.56% in the Colloid T group (*p* < 0.05) and 24.67 ± 3.18% in the Colloid V group (*p* < 0.05) *vs*. 19.10 ± 2.68% in the Crystalloid group]. There was no difference between the two Colloid groups on day 7 (Fig. 1).

**Figure 2:**
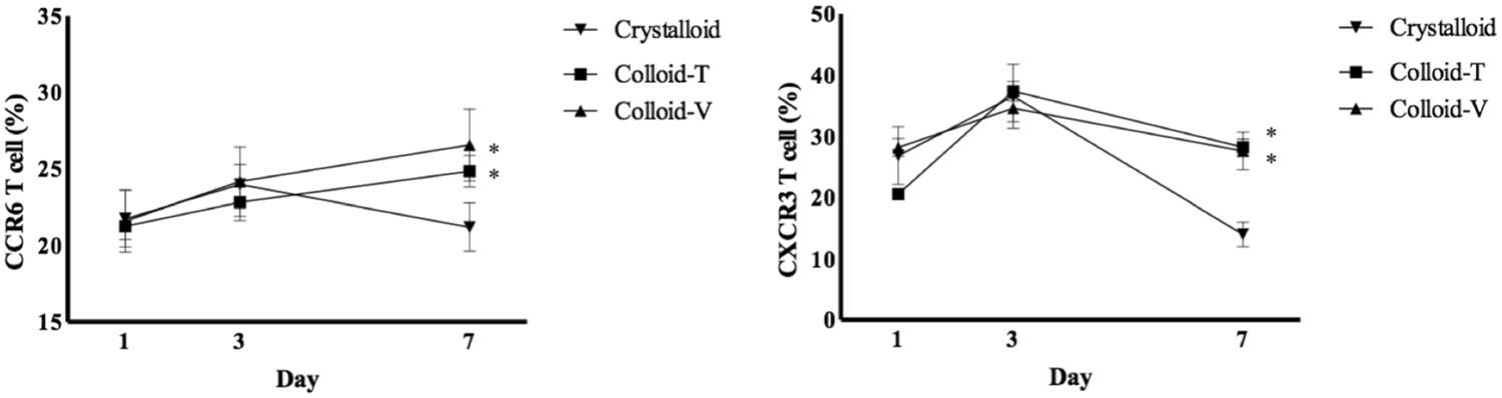
The chemokines for immune cells. Abbreviations: CCR6, CC-type chemokine 6; CXCR3, CXC chemokine receptors 3.

The chemokine levels also did not differ among the three groups on days 1 and 3. On day 7, however, the chemokine levels were significantly higher in the Colloid-T and Colloid-V groups than the Crystalloid group [frequency of CCR6: 25.96 ± 2.26% in the Colloid T group (*p* < 0.05) and 27.74 ± 3.80% in the Colloid V group (*p* < 0.05) *vs*. 20.76 ± 2.99% in the Crystalloid group; frequency of CXCR3: 28.18 ± 3.00% in the Colloid T group (*p* < 0.05) and 27.58 ± 6.89% in the Colloid V group (*p* < 0.05) *vs*. 14.00 ± 4.48% in the Crystalloid group]. There was no difference between the two Colloid groups on day 7 (Fig. 2).

The cytokine levels differed significantly at all times, except TNF-α on day 1, between the Crystalloid group and Colloid groups (Table 2), while there were no differences between the two Colloid groups at any time point.

**Table 2:** Cytokines for immune cells.

Parameter	Day	Crystalloid group	Colloid-T group	Colloid-V group
IFN-γ (ng/mL)				
	Day 1	13.72 ± 3.21	23.11 ± 6.23^*^	24.17 ± 5.20^*^
	Day 3	15.54 ± 2.13	35.20 ± 1.27^*^	34.59 ± 3.64^*^
	Day 7	17.38 ± 3.16	35.51 ± 2.31^*^	46.38 ± 3.90^*^
IL-2 (ng/mL)				
	Day 1	9.86 ± 1.40	19.53 ± 1.31^*^	19.22 ± 2.57^*^
	Day 3	11.25 ± 5.30	25.27 ± 2.75^*^	28.21 ± 3.73^*^
	Day 7	9.22 ± 0.91	27.44 ± 3.63^*^	27.15 ± 4.24^*^
TNF-α (pg/mL)				
	Day 1	304.33 ± 102.70	204.85 ± 50.11	315.73 ± 78.31
	Day 3	56.14 ± 11.46	180.47 ± 39. 66^*^	150.27 ± 48.11^*^
	Day 7	48.26 ± 8.68	128.42 ± 22.10^*^	118.47 ± 27.36^*^

Data are expressed as mean ± standard deviation.

Abbreviations: IFN-γ, interferon-gamma; IL-2, Interleukin-2; TNF-α, tumor necrosis factor (TNF)-α.

^*^p < 0.05 compared with Crystalloid group.

**Figure 3:**
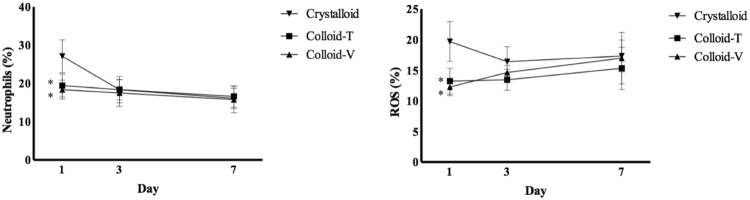
The expression of neutrophil with reactive oxygen species (ROS). Abbreviations: reactive oxygen species, ROS. ^*^p < 0.05 compared with Crystalloid group.

The frequency of neutrophils producing ROS was significantly lower on day 1 in the two synthetic Colloid groups compared with the Crystalloid group [neutrophils: 19.44 ± 3.15% in the Colloid T group (*p* < 0.05) and 18.39 ± 2.50% in the Colloid V group (*p* < 0.05) *vs*. 27.10 ± 4.51% in the Crystalloid group; ROS: 13.22 ± 2.10% in the Colloid T group (*p* < 0.05) and 12.27 ± 1.43% in the Colloid V group (*p* < 0.05) *vs*. 19.70 ± 3.26 in the Crystalloid group]. However, the values on the other days were similar in all three groups (Fig. 3).

## Discussion

After co-culture with mouse splenocytes, there were significantly more CD4^+^ and CD8^+^ T cells in the synthetic Colloid groups, compared with the Crystalloid group on day 7. The chemokine levels showed patterns similar to the frequencies of CD4^+^ and CD8^+^ T cells. The cytokine levels were significantly higher in the synthetic Colloid groups at all times compared with the Crystalloid group. The frequency of neutrophils producing ROS was significantly lower in the two synthetic Colloid groups on day 1 compared with the Crystalloid group.

Although there is an association between renal injury and hyperchloremic acidosis after the use of non-balanced synthetic colloid^8,9^, balanced synthetic colloid is still popularly used. However, it is not recommended as the first choice for sepsis because of the risk of renal injury^10^. Since the definite cause of renal injury after the use of synthetic colloid in sepsis is not known, studies of the effects of fluid on the immune response might provide a clue.

We focused on T cells to evaluate the immune response. During fluid therapy, the body recognizes the infused fluid as a foreign body, and an immune response occurs. The acquired immune response involving T cells follows the innate immune response, involving neutrophils. T cells have recently been spotlighted as a bridge between the innate and acquired immune responses against cell injury and organ dysfunction^11^.

Several studies have investigated the effects of synthetic colloid on neutrophils. Handrigan *et al*. showed that synthetic colloid inhibited neutrophil adhesion and trans-endothelial migration^12^. Matharu *et al*. demonstrated that synthetic colloid inhibited neutrophil recruitment during ischemia–reperfusion injury. This was associated with reduced expression of adhesion molecules and chemokines^13^. Rossaint *et al*. demonstrated that synthetic colloid significantly reduced neutrophil–platelet aggregates, neutrophil extracellular trap formation, chemokine-induced arrest, and neutrophil transmigration under inflammatory conditions^14^. In the present study, the frequency of neutrophils was reduced in the synthetic Colloid groups compared with the Crystalloid group. However, a significant difference was seen only on day 1. This finding is remarkable. An innate immune response occurs after exposure to pathogens, and the immediate response is the strongest. In comparison, there is a delay in attaining the maximal acquired immune response after exposure to the pathogen. Therefore, we checked the immune response until day 7. The depressed neutrophil activity in the two synthetic Colloid groups on day 1 indicates that the synthetic colloid inhibited the host innate immune response. Wiedermann stated that the reduced neutrophil activity caused by synthetic colloid is not beneficial. The reduced neutrophil recruitment, migration, and neutrophil–platelet interactions indicate limited neutrophil-dependent defenses^15^. Moreover, the depressed neutrophil activity in the two synthetic Colloid groups on day 1 was not associated with the decrease in the acquired immune response.

In the present study, we evaluated the changes in the frequencies of immune cells following fluid administration. The effect of the synthetic colloid on the immune response is summarized below, although further evaluation is required. The hydroxyethyl starch in the synthetic colloid was recognized as a foreign body and induced an immune response, while crystalloid did not. Initially, the synthetic colloid reduced neutrophil recruitment, migration, and neutrophil–platelet interaction. The effects of the synthetic colloid on the host resulted in a limited neutrophil-dependent innate immune response. Finally, T-cell activation as part of an acquired immune response was increased by the increased levels of chemokines.

The increase in the acquired immune response in the synthetic Colloid groups, including elevation of CD4^+^ and CD8^+^ T cell numbers, is likely associated with cell injury and organ dysfunction, compared with the innate immune response involving neutrophils. T cells are used as a marker of diagnostic and therapeutic strategies for renal injury^16,17^. However, neutrophil depletion does not protect against acute kidney injury^18,19^, although increased neutrophil numbers were observed, and their reduction is beneficial in acute kidney injury^20^.

This study had several limitations. First, this was an *in vitro* study involving co-cultured mouse splenocytes, and we did not postulate a mechanism for the results. Our findings suggest that crystalloid and synthetic colloids have different effects on immune cells, but the results should be interpreted with caution. Second, we evaluated only balance colloids. Although the aim was to assess the effect of hydroxyethyl starch on immune cells, evaluation of non-balanced colloids is required to confirm the results. Third, we did not assess the effect of buffer components used to generate bicarbonate; *e*.*g*., acetate, gluconate, and malate. These compounds are known to modulate the host immune response, although the magnitude of their effects is limited.

In conclusion, crystalloid had a limited effect on the immune response. On the other hand, synthetic colloid increased the acquired immune response, although it temporarily inhibited the innate immune response.
